# Impact of Pentoxifylline and Vitamin E on Ribavirin-Induced Haemolytic Anaemia in Chronic Hepatitis C Patients: An Egyptian Survey

**DOI:** 10.4061/2011/530949

**Published:** 2011-05-14

**Authors:** M. Assem, M. Yousri

**Affiliations:** Departments of Hepatology and Gastroenterology, National Liver Institute, Monoufiya University, Sheben Al kom 3211, Egypt

## Abstract

*Background/Aim*. We evaluate the impact of combined pentoxifylline and high-dose vitamins E to standard antiviral treatment on RBV-induced haemolytic anaemia. *Patients and Methods*. Selected 200 naïve chronic HCV patients, were randomized to receive either the standard antiviral therapy (peginterferon *α*-2b and RBV) plus pentoxifylline (800 mg) and high-dose vitamin E (1000 iu) daily (combined group) or received standard antiviral therapy plus placebo only (control group). They were followed up during treatment course and for 6 months posttreatment to assess the occurrence of anaemia and virological response, respectively. *Results*. RBV dose modification due to anaemia were significantly less in combined group (8.5 versus 21.5%. *P* < .05).Withdrawal, secondary to sever anemia (Hb < 8.5 gm%), was recorded only in 6 (28.6%) patients of the control group. Both (ETR) and (SVR) were significantly higher in combined group than control group by both intention-to-treat analysis (71 versus 56%, *P* < .05 and 66 versus 49%, *P* < .05) and per-protocol analysis (85.5 versus 70.9%, *P* < .05 and 79.5 versus 62%, *P* < .05). *Conclusion*. Pentoxifylline and vitamin E can ameliorate RBV-associated haemolysis; improve compliance and virologic clearance when combined with the standard antiviral therapy in patients with chronic hepatitis C.

## 1. Introduction

The hepatitis C virus (HCV) is a major public health problem and a leading cause of chronic liver disease, with approximately 3% of the world's population affected [1, 2]. Hepatitis C genotype 4 is the prevalent genotype in the Middle East [3]. Its prevalence in Egypt is estimated to be more than 90% of chronic hepatitis C patients [4]. 

Recently, ribavirin (RBV) has been used in combination with pegylated interferon (IFN) as the most effective antiviral therapy [5]. However, RBV-induced anaemia is the major dose-limiting factor in the combined therapy of RBV and IFN products [6]. 

Anaemia is likely related to extensive RBV accumulation in erythrocytes subsequent to possible active unidirectional transmembrane transport. RBV exerts its toxicity through the inhibition of intracellular energy metabolism and oxidative membrane damage, leading to an accelerated extravascular hemolysis by the reticuloendothelial system [7].

Previous studies proved that the maintenance of high-dose ribavirin by preventing haemolytic anaemia using high supplementation doses of antioxidants, like vitamin E and vitamin C, may lead to better sustained virological response rate by pegylated IFN plus ribavirin therapy, indicating that the dose of ribavirin is an important factor for effective combination therapy [8]. 

Pentoxifylline which is a trisubstituted xanthine derivative has been used successfully in the treatment of peripheral arterial disease [9], diabetic nephropathy [10], diabetic retinopathy [11], and coronary artery disease in diabetics [11], generally in all conditions in which erythrocyte stiffness and increased blood viscosity are believed to play a role [11]. Recently, it showed many advances in liver diseases [12]. It has long been viewed as a potential antifibrotic agent [13], in addition to its modulating effect on insulin resistance through downregulation of TNF, it could be a potential mechanism for improvement in patients with nonalcoholic steatohepatitis (NASH) [12, 14, 15]. Our study aimed to evaluate the impact of combined pentoxifylline and high-dose vitamin E on the standard treatment of HCV to try ameliorating ribavirin-induced anaemia, aiming to improve treatment adherence and virological response.

## 2. Patients and Methods

### 2.1. Patients' Selection

This is a prospective, randomized, single-blind, controlled study, carried out at the National Liver Institute, Menoufiya University, Sheben AL koom, Egypt. The institute was established to provide specialized care for patients with liver disease. The center receives patients from almost all regions of Egypt, besides patients from the neighbouring Arab countries.

Between January and May 2008, four hundred and eleven chronic hepatitis C patients presented to the outpatient clinics, and they were examined and screened for the eligibility of HCV treatment. They were CHC-genotype 4 Egyptian patients of either sex with age ranged from 18 to 50 years and had detectable HCV RNA and liver biopsy consistent with chronic hepatitis and significant fibrosis. 

A total of 188 patients were excluded from the trial because of concurrent infection with hepatitis B (38 patients), presence of hepatocellular carcinoma (8 patients), decompensated cirrhosis (11 patients), history of psychiatric troubles (18 patients), presence of baseline anaemia ((Hb < 13 g/dL for men and <12 g/dL for women)—39 patients), low absolute neutrophil count (ANC) <1500/mm^3^ (16 patients), and low baseline platelet count <75,000/mm^3^ (58 patients). An additional 23 patients declined to participate in the study.

This study complies with the standards of the Declaration of Helsinki and current ethical guidelines. After institutional review board approval and written informed consent were obtained, 200 subjects were recruited for the study. These were randomly assigned into two equal groups with the aid of a computer-generated table of random numbers.

### 2.2. Treatment Regimens

Each group received standard antiviral therapy, includingp peginterferon *α*-2 b ((1.5 mcg/kg/week) (Pegintern, Schering-Plough Co., Kenilworth, NJ, USA)) plus ribavirin (Rebetol, Schering-Plough Co., Kenilworth, NJ, USA) in dose of 1000 or 1200 mg/day depending on patient weight if less or more than 75 kg, respectively. The duration of treatment was assigned for 48 weeks.

In the combined group, patients received high dose of vitamin E (Vitamin E, Pharco Co., Egypt) in a dose of 1000 IU/day plus pentoxifylline (Trental 400 SR, product of Aventis Pharma, Germany) in a dose of 800 mg/day orally, in addition to standard antiviral therapy, while patients in the other group received starch tablets (placebo) plus the standard antiviral therapy, and they were considered as a control group. All medications were supplied by the National Liver Institute, Egypt.

### 2.3. Dose Modification

Medications doses were modified if haematological adverse events had occurred according to the guidelines in Table 1 [16]. Growth factors such as erythropoietin and granulocyte colony-stimulating factor (G-CSF) were not given in this study except when patients were beyond the dose modification criteria, and those would be ruled out from the study.

### 2.4. Followup and End Points

All subjects were monitored during peginterferon-ribavirin treatment in both groups and for a further period of 24 weeks after the end of treatment. They had biweekly outpatient visits during the first month and monthly visits during the rest of the treatment period as well as during the 24-week follow-up period. At each visit, a physical examination was performed, treatment and the importance of treatment adherence were explained, adverse effects were recorded, and biochemical tests and blood counts were performed. The efficacy to standard antiviral therapy was defined as undetectable HCV RNA level by PCR at 12 weeks (early virological response EVR), at the end of treatment (ETR), and after 24-week followup (SVR).

The primary end point of our study was to complete the study protocol without major dose reduction due to anemia, while secondary end points were the occurrence of treatment complication necessitating withdrawal or antiviral treatment failure.

### 2.5. Statistical Analysis

Data was statistically analyzed using SPSS (Statistical Package for Social Science) program version 17 for windows. Quantitative data were presented as mean ± SD. Qualitative data are presented as relative proportions. Student *t*-test and the Mann-Whitney test were used to compare's means and medians for quantitative variables where appropriate. The association between the categorical variables was assessed by using the Chi-square test or Fisher Exact test. *P* < .05 was considered as statistically significant.

## 3. Results

### 3.1. Basal Patients' Profile and Laboratory Data

Demographic features, laboratory, and histological staging in both studied groups were presented in Table 2. There was no statistical difference between both groups as regards mean age, sex ratio, mean body mass index, basal hematological features, and blood chemistry.

As regards histological finding, there was no statistical difference between patients with early fibrosis (F1,F2) and those with advancing fibrosis (F3,F4) in both studied groups (*P* > .05). Also, there was no statistical difference as regards the number of patients receiving initial ribavirin dosage (either 1000 or 1200 mg/day) between both groups (*P* > .05).

### 3.2. Efficacy and Safety

A number of patients who completed the study protocol (48 ws) were comparable between both studied groups (83% in combined group versus 79% in control group, *P* > .05) (Table 3). In combined group, 66 patients (79.5%) completed the protocol without dose modification of either peginterferon or ribavirin, while 7 patients (8.4%) needed ribavirin dose modification due to the occurrence of mild anemia (Hb < 10 gm, but >8.5 gm%). In addition, in 5 patients (6%) both drugs were reduced due to combined anemia, neutropenia, and thrombocytopenia (Table 3).

Seventeen patients (17%) in the combined group were dropped out of the study; none of them had hematological complications (Table 4). No complications related to either vitamin E (heart failure, coagulopathy, and gastrointestinal effects) or pentoxifylline (cardiac arrhythmia, dizziness, and gastrointestinal effects) were recorded during study protocol, except some cases with mild flushing and/or headache not necessitating dose modification or discontinuation.

In the control group, 54 patients (68.4%) completed study protocol without dose modification of both drugs, while 17 patients (21.5%) needed ribavirin dose modification due to the occurrence of mild anemia, and another 4 patients (5%) needed reduction of both drugs due to combined blood cytopenias.

Twenty-one patients (21%) in the control group were dropped out of the study, with anemia as the cause of discontinuation in 6 patients (28.6%) (Table 4). Patients who were dropped out from hematological complications in control group were ruled out of the study protocol with receiving the appropriate treatment

### 3.3. Hematological Changes: (Figures 1, 2, and 3)

As regards the changes in hematological profile during treatment protocols, combined group showed significantly less reduction of hemoglobin, leucocytes, and platelets if compared to control group (*P* = .01, *P* = .009, and *P* = .01, resp.).

### 3.4. Virological Response

Both ETR and SVR were significantly higher in the combined group than those of the control group by both intention to treat analysis (71 versus 56%—*P* < .05 and 66 versus 49%, *P* < .05) and per protocol analysis (85.5 versus 70.9%, *P* < .05 and 79.5 versus 62%, *P* < .05) (Table 5).

## 4. Discussion

In this study, we elucidated, for the first time, that the combination pentoxifylline and vitamin E significantly ameliorates the reduction of hemoglobin level during treatment with peginterferon and ribavirin in chronic hepatitis C Egyptian patients with significant improvement in both ETR and SVR rates in a randomized controlled trial.

Clinical evidence has shown the importance of ribavirin in the treatment of CHC patients, but this agent is associated with frequent adverse events, necessitating dose reductions and/or discontinuations [17]. Ribavirin reductions, however, can have a negative impact on SVR. Thus, the management of ribavirin toxicity, especially anaemia, can allow patients to continue full-dose combination therapy with peginterferon and ribavirin, aiming for enhancing their probability of attaining SVR [18].

Previous studies showed that vitamin E might protect red blood cell membranes from oxidative damage due to ribavirin [19]. However, while many advocate the potential benefits of antioxidants, including vitamin E, on ribavirin-induced haemolysis, no systematic studies have been reported [20].

Based on previous reports of pentoxifylline in increasing erythrocyte adenosine triphosphate levels and erythrocyte deformability [21], and decreasing plasma fibrinogen and blood viscosity [21] which lead to reduction of both hemolysis and pain crises in sickle cell disease [22], we assumed that the combination of pentoxifylline and vitamin E may be more effective in ribavirin-induced haemolytic anaemia. 

In our study, the addition of pentoxifylline and vitamin E to peginterferon and ribavirin improved reduction in haemoglobin, more significantly than the other group who took antiviral therapy plus placebo. This was similar to the results of a previous work done by Kawaguchi et al., who cleared that the addition of high doses of vitamin E and vitamin C could prevent haemolytic anaemia during combination therapy with ribavirin and IFN *α*-2b in patients with chronic hepatitis C [19], while Saeian et al. recommended the addition of another water-soluble antioxidant, like N-acetyl cysteine to vitamin E, aiming to enhance its antioxidant protective effect on erythrocyte membrane [20].

In our study, the protective impact of the combined therapy was seen through the reduction of both neutrophils and platelet counts during treatment period which may point to the extended protective effect of this combination to prevent reticuloendothelial system (RES) destruction for different blood cells. It is suggested that pentoxifylline may increase the filterability of different blood cells while passing through RES based on its proven role to increase filterability of red blood cells and decrease adherence to endothelial cells [21].

A similar finding was reported by another group [19] concerning the protective effect of antioxidants like vitamin E and vitamin C on platelet counts. They attribute their results just to antioxidant proprieties of the used vitamins. 

The number of patients needed ribavirin dose reduction was significantly less in the combined group which reflects the protective role of adding pentoxifylline and vitamin E to standard treatment of HCV. This improved patients' compliance and adherence to treatment was associated with significant improvement of both ETR and SVR rates in the combined group, which highlighted the beneficial effect of this combination in increased compliance, maintained high dose of ribavirin, and may be the inhibitory effect of pentoxifylline on several proinflammatory cytokines in the liver like TNF-*α* [23], which might augment the antiviral role of peginterferon and ribavirin.

Withdrawal from the study was comparable between studied groups. Anemia was the most significant cause of withdrawal in control group, while no specific complication related to either pentoxifylline or vitamin E was recorded. This reflects the safety of this combination when added in the used conditions to the standard treatment of HCV during the treatment protocol. 

Previous reports denoted that administering erythropoietin can improve anaemia caused by peginterferon and ribavirin therapy and is more effective than dose reduction at improving quality of life during treatment [24]. However, erythropoietin, which is not approved by the US Food and Drug Administration (FDA) for the use in patients with HCV infection, adds another parenteral drug to the patient's treatment regimen and is associated with additional costs, inconvenience, and potential side effects [24], while new suggested combination is safe and cost not more than 50 dollar/treatment course for 48 wees.

We concluded that adding both pentoxifylline and vitamin E could ameliorate ribavirin-associated haemolysis, improving compliance and virologic clearance with combination therapy with pegylated interferon and ribavirin in patients with chronic hepatitis C. The suggested combination was cheap, safe, and effective and could replace the use of more hazards and expensive erythropoietin in our studied population, so worldwide use of this combination is recommended to verify their efficacy and safety in other genotypes of HCV patients.

##  Conflict of Interests

Authors declare no conflict of interests, either financial or nonfinancial.

##  Funding

National Liver Institute participated in the fund.

## Figures and Tables

**Figure 1:**
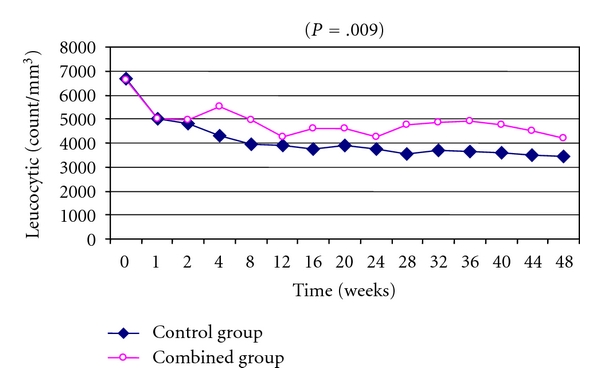
Time course changes in mean leucocytic counts among the studded groups showed significant improvement in leucocytic reduction in combined group during study protocol (*P* = .009).

**Figure 2:**
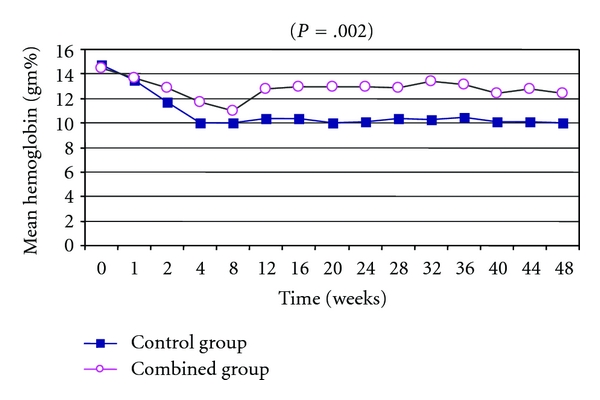
Time course changes in mean haemoglobin percentage among the studded groups showed significant improvement in haemoglobin reduction in combined group during study protocol (*P* = .002).

**Figure 3:**
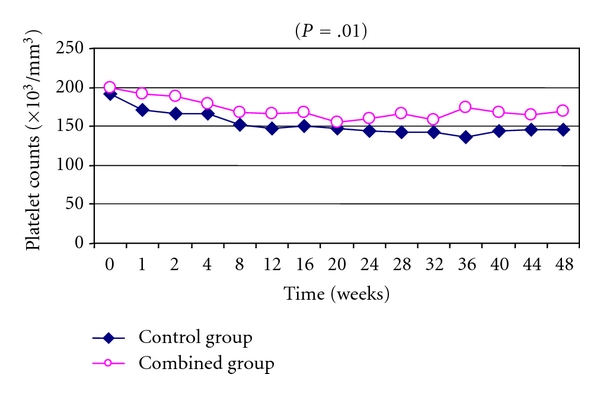
Time course changes in mean platelet counts among the studded groups showed significant improvement in platelet count reduction in combined group during study protocol (*P* = .01).

**Table 1:** Guidelines for dose modification and discontinuation of PEG-Intron or PEG-Intron/Rebetol for Hematologic toxicity [16].

Laboratory values PEG	PEG-Intron	Rebetol
Hb*		
<10.0 g/dL	—	Decrease by 200 mg/day
<8.5 g/dL	Permanently discontinue	Permanently discontinue
WBC		
<1.5 × 10^9^/L	Reduce dose by 50%	—
<1.0 × 10^9^/L	Permanently discontinue	Permanently discontinue
Neutrophil		
<0.75 × 10^9^/L	Reduce dose by 50%	—
<0.5 × 10^9^/L	Permanently discontinue	Permanently discontinue
Platelets		
<80 × 10^9^/L	Reduce dose by 50%	—
<50 × 10^9^/L	Permanently discontinue	Permanently discontinue

*For patients with a history of stable cardiac disease receiving PEG-Intron in combination with ribavirin, the PEG-Intron dose should be reduced by half and the ribavirin dose by 200 mg/day if a >2 g/dL decrease in haemoglobin is observed during any 4-week period. Both PEG-Intron and ribavirin should be permanently discontinued if patients have haemoglobin levels <12 g/dL after this ribavirin dose reduction.

Hb: haemoglobin

WBC: White blood cell.

**Table 2:** Comparison of patient profiles and laboratory data in both groups.

Variable	Group 1 (vit E and pentoxifylline) *n* : 100	Group 2 (control) *n* : 100	*P* value
Age (years)	44.3 ± 12.3	45.9 ± 10.3	NS
Sex M : F	69 : 31	58 : 42	NS
BMI kg/m^2^	26.8 ± 5.7	27.9 ± 6.1	NS
Peripheral blood cell count			
Haemoglobin gm%	14.48 ± 1.64	14.79 ± 1.38	NS
Leukocyte (×10^3^/mL)	6.64 ± 1.85	6.67 ± 1.9	NS
Platelet (×10^3^/mL)	198.73 ± 62.4	192.21 ± 76.32	NS
Blood biochemistry			
AST (IU/L)	72.55 ± 46.82	73.19 ± 39.47	NS
ALT (IU/L)	86.71 ± 67.92	79.66 ± 70.13	NS
Bilirubin (mg/dL)	0.91 ± 0.62	1.02 ± 0.66	NS
Creatinine (mg/dL)	1.13 ± 0.42	0.99 ± 0.38	NS
A.P. (IU/L)	124.3 ± 54.82	132.02 ± 64.7	NS
Iron (mcg/dL)	82.7 ± 60.7	85.6 ± 58.8	NS
Ferritin ng/mL	132.7 ± 42.8	129.64 ± 39.24	NS
Liver Histology			
F1-F2	68	62	NS
F3-F4	32	38	
HCV-RNA PCR viral load iu/mL			
<400,000 iu/mL	66	58	NS
>400,000 iu/mL	34	42	
Ribavirin dosage			
1000 mg/day	38	29	NS
1200 mg/day	62	71	

NS: Non significant (*P* > .05).

**Table 3:** Course of antiviral treatments in studied groups.

Variable	Combined group	Control group
No and % of patients who completed the study protocol		
With full dose of both drugs	66	54
Ribavirin reduction	7	17
Interferon reduction	5	4
Reduction of both drugs	5	4
No and % of patients who discontinued the study protocol		
With dose reduction	17	21

**Table 4:** Causes of dropout from study protocols among studied groups.

Variable	Combined group *n* : 17	Control group *n* : 21	Significance
Thyroiditis	2	2	NS
Autoimmune disease aggravation	2	1	NS
Skin eruption	3	1	NS
Depression or anxiety	3	2	NS
Fatigue	2	1	NS
Anaemia Hb < 8.5 gm%	—	6	S
Neutropenia ANC < 500/mm^3^	—	3	S
Thrombocytopenia Platelet < 25 × 10/mm^3^	—	2	NS
Retinal haemorrhage	1	—	NS
Nephritic syndrome	—	1	NS
Hearing disturbance	1	1	NS
Economical problems	3	1	NS

S: significant (*P* < .05)

NS: nonsignificant (*P* > .05).

**Table 5:** Comparison of different virological responses between studied groups.

Response	Studied groups		Chi-square test	*P* value
	Combined	Control		
	Group 1	Group2		
Intention to treat	*n* : 100	*n* : 100		
ETR	71 (71%)	56 (56%)	4.854	0.027*
SVR	66 (66%)	49 (49%)	5.913	0.015*
Per protocol	*n* : 83	*n* : 79		
ETR	71 (85.5%)	56 (70.9%)	5.133	0.023*
SVR	66 (79.5%)	49 (62%)	6.014	0.014*

ETR: end-of-treatment response.

SVR: sustained virological response.

*significant.
